# Healthy Eating Vital Sign: A New Assessment Tool for Eating Behaviors

**DOI:** 10.5402/2012/734682

**Published:** 2012-07-22

**Authors:** Jessica L. J. Greenwood, Junji Lin, Danita Arguello, Trever Ball, Janet M. Shaw

**Affiliations:** ^1^Division of Public Health, Department of Family and Preventive Medicine, University of Utah, 375 Chipeta Way, Ste A, SLC, UT 84108, USA; ^2^Pharmacotherapy Outcomes Research Center, University of Utah, 421 Wakara Way, Suite 208, SLC, UT 84112, USA; ^3^Department of Exercise and Sport Science, University of Utah, SLC, UT 84112, USA

## Abstract

*Introduction*. Most dietary questionnaires are not created for use in a clinical setting for an adult health exam. We created the Healthy Eating Vital Sign (HEVS) to assess eating behaviors associated with excess weight. This study investigated the validity and reliability of the HEVS. *Methods*. Using a cross-sectional study design, participants responded to the HEVS and the Block Food Frequency Questionnaire (BFFQ). We analyzed the data descriptively, and, with Pearson's correlation and Cronbach coefficient alpha. *Results*. We found moderate correlation (rho > 0.3) between multiple items of the HEVS and BFFQ. The Cronbach's alpha was 0.49. *Conclusion*. Our results support the criterion validity and internal reliability of the HEVS as compared to the BFFQ. The HEVS can help launch a dialogue between patients and providers to monitor and potentially manage dietary behaviors associated with many chronic health conditions, including obesity.

## 1. Introduction

The United States (US) did not meet the *Healthy People 2010* objective to decrease the prevalence of adult obesity to 15% [1]. According to the Centers for Disease Control and Prevention (CDC), 27% of US adults are obese [2]. Flegal et al. report more dismal statistics; 68% of adults are overweight (BMI ≥ 25) and 34% are obese (BMI ≥ 30) [3].

Multiple factors influence energy balance, or energy intake versus energy expenditure. However, primary care providers have the opportunity to engage with people and potentially affect behaviors that can tilt the energy balance [4]. A provider's attention to weight has great influence on patients [5, 6]. Therefore, effectively and efficiently managing and preventing overweight and obesity in the primary care setting is warranted [7].

Traditionally, food frequency questionnaires are used to assess habitual dietary behavior. The Block Food Frequency Questionnaire (BFFQ) is a valid and reliable instrument used as a standard tool for nutrition assessment [8, 9]. The BFFQ contains more than 100 questions, takes approximately 45 minutes to complete, and focuses on nutrient intake rather than eating behaviors. Because of its length and the complexities involved in dietary analysis, the BFFQ is too cumbersome for routine clinical screening of patients' nutritional habits. Recognizing this problem, Glasgow et al. recommended Starting the Conversation-(STC) Diet and the Summary of Diabetes Self-Care Activities (SDSCA) as practical measures for clinical use [10]. The STC-Diet is a 7-item instrument to assess dietary behaviors, created for the New Leaf (Well-Integrated Screening and Evaluation for Women in Massachusetts, Arizona and North Carolina) (WISEWOMAN) intervention program [11]. To our knowledge, this scale has not been studied for validity and reliability. The SDSCA is an 11-item survey that assesses many health indicators for diabetic patients. It includes two questions of specific foods eaten in the last 7 days: fruits and vegetables, and high fat foods [12]. These questionnaires, however, were not created for use in a clinical setting for a routine adult health exam.

Our previous review indicated five specific eating behaviors associated with excessive weight status: (1) frequent fast food/restaurant consumption, (2) consumption of large portion sizes, (3) high consumption of sugar-added beverages, (4) low fruit and vegetable consumption, and (5) infrequent breakfast consumption [13]. We created the Healthy Eating Vital Sign (HEVS) to clinically address these specific eating behaviors as risk factors for obesity, which, to our knowledge, no other tool addresses in a clinical setting. The HEVS questions recent, one-day or one-week, recall of eating behaviors, *and* typical behavior over a day or a week. For example, one of the recent recall questions of the HEVS is “How many times did you drink juice or punch *yesterday*?” One of the HEVS typical recall questions is “How many times do you typically eat vegetables in *one day*?” (see the Appendix).

Previous evaluation supported the content validity of the HEVS. We found that sugar-added beverage consumption (*β* = 0.61, *P* = 0.006) and consuming full portion sizes served at restaurant/fast food establishments (OR = 1.34, *P* = 0.005) were positively associated with BMI and obesity, while eating at restaurant/fast food establishments (OR = 1.24, *P* = 0.005) was positively associated with being overweight. Consumption of more than 3 fruits and/or vegetables a day tended to have a negative association with BMI (*β* = −0.09, *P* = 0.66); however, this behavior is associated with weight stability [14]. After examining its content validity, the HEVS was revised for the present study, eliminating the questions regarding breakfast and consumption of large portion sizes. We deemed our question of breakfast consumption too vague for adequate correlation with weight. Because large portion size has been closely associated with restaurant and fast food consumption, we excluded the question of large portion size [15]. 

Utilizing a cross-sectional study design, the purpose of this investigation was to determine the criterion validity and internal reliability of the HEVS. Our ultimate goal is to develop a screening tool for use in primary care clinics that would help providers identify these potentially modifiable behaviors among patients.

## 2. Methods

The Institutional Research Review board of The University of Utah approved this research with human subjects (IRB no. 00038314).

### 2.1. Clinic Selection

To further explore the HEVS, we joined investigators of the Utah Women's Health Information Network (UWIN). UWIN reflects partnerships among the University of Utah, the Utah Department of Health, the Association for Utah Community Health, and Utah Navajo Health Services, Inc. One UWIN project aimed to validate relatively new assessments of physical activity and nutrition that can be used in primary care clinics. The HEVS was added to this project. 

### 2.2. Participant Selection

Participants were recruited from 8 primary care clinics. The UWIN grant partnership facilitated data collection among four, federally qualified community health centers (Community Health Centers (CHCs), Inc.). The remaining 4 clinics were participants of the Utah Health Research Network (UHRN), which includes 10 clinics within the University of Utah Health Care Community Clinics system. These four clinics were recommended by the study coinvestigators and selected based on their similarity to the participating CHC Inc. sites.

We recruited clinic staff (i.e., medical assistants, medical records, and billing specialists and nurses) of the targeted CHC, Inc. and UHRN sites. We invited women and men ≥ 18 years to participate. Potential participants with uncontrolled chronic disease, significant musculoskeletal disease that would limit physical activity, or women who were pregnant were excluded. 

### 2.3. Procedures

We communicated with clinic administration to schedule times for recruitment and data collection. Research personnel visited each clinic on two occasions. On Day 1, we reviewed the study, determined participant eligibility, obtained consent, explained the BFFQ, and conducted basic demographic and health history assessments. The health history was self-reported, including height and weight. Depending upon participant self-identification of typical dietary habits, some received the version of the BFFQ that is based upon the Hispanic-American Diet, while others were given the BFFQ based upon the typical American diet [16]. Although the BFFQ uses different food lists to accommodate Hispanic and typical American nutritional habits, they provide the same dietary analysis. The participants took the BFFQ home with them for completion.

Day 2 of data collection occurred one week after the first, whereupon participants returned their completed BFFQ and completed the HEVS. Research personnel checked the BFFQ for missing data and then administered the HEVS questionnaire verbally and circled participants' answers. HEVS required about 1 minute to administer for most participants.

### 2.4. Analysis

We calculated the BMI utilizing the formula BMI = weight/(height^2^)∗703. We used SAS/STAT Software for analysis. We defined moderate correlation as rho = 0.3–0.6, and significance at *P* ≤ 0.05. We also calculated the cronbach coefficient Alpha to indicate the internal reliability of HEVS. We defined alpha > 0.4 as acceptable [17].

## 3. Results

Participants (*N* = 60, 38.3 ± 9.6 yrs.) were mostly female (*n* = 56). Fifty-four percent reported to be White/Caucasian, and 25% were Latino/a. All participants were employees of the CHC, Inc. or UHRN Clinics. Thirty-two percent had a normal BMI (19 < BMI < 25), 25% were overweight (30 < BMI > 25), and 43% were obese (BMI > 30).  Table 1 describes other participant demographics. All participants completed the HEVS and the BFFQ.  Table 2 provides a numerical summary of the HEVS and BFFQ responses.

Pearson's correlation analysis revealed moderate association between multiple items of the HEVS and the BFFQ (see Table 3). Most significant associations were between the number of times participants ate at restaurant or fast food establishments and BFFQ responses to fat grams (recently and typical, *r* = 0.3, *P* < 0.05), and total trans-fat grams (recently, *r* = 0.5, *P* < 0.001; typical, *r* = 0.4, *P* < 0.01), and daily servings of bread, rice, pasta, and cereal (recently, *r* = 0.4, *P* < 0.01; typical, *r* = 0.3, *P* < 0.05). 

The HEVS recall of typical consumption of non-diet soda was most significantly associated with BFFQ responses to total kilocalories of energy (*r* = 0.4, *P* < 0.01), total grams of sugar (*r* = 0.4, *P* < 0.01), and percentage of kilocalories from sweet and desserts (*r* = 0.3, *P* < 0.05). Recent and typical HEVS recall responses from the number of times participant drank juice or punch were moderately associated with BFFQ responses to total kilocalories of energy (recently and typical, *r* = 0.3, *P* < 0.05), and total grams of sugar (recently and typical, *r* = 0.4, *P* < 0.01).

Recent and typical HEVS recall responses from the number of times participants ate vegetables were moderately associated with BFFQ responses to overall dietary fiber grams (recently and typical, *r* = 0.3, *P* < 0.05), dietary fiber grams from fruits and vegetables (recently and typical, *r* = 0.4, *P* < 0.01), daily servings of vegetables (recently and typical, *r* = 0.3, *P* < 0.05), and daily frequency of fruits and fruit juices (*r* = 0.4, *P* < 0.0.1 and 0.3, *P* < 0.05). The typical responses from the HEVS question of vegetable consumption were negatively correlated with the total trans-fat grams (*r* = 0.3, *P* < 0.05).

Recent and typical HEVS responses from the number of times participants ate fruits were moderately associated with BFFQ responses to overall dietary fiber grams (recently and typical, *r* = 0.3, *P* < 0.05), and daily frequency of fruits and fruit juices (recently and typical, *r* = 0.4, *P* < 0.01). 

The standardized Cronbach alpha for internal consistency was 0.49 (see Table 4).

## 4. Discussion

This investigation demonstrates evidence for criterion validity and internal reliability of the HEVS. Our results support the criterion validity of the HEVS as compared to the BFFQ. Most of the significant correlations would be classified as moderate in strength. Further, most of the results demonstrate correlations among like nutritional variables; for example, the HEVS fruit and vegetable consumption questions were correlated primarily with BFFQ fruit and vegetable variables. The associations between HEVS responses to the consumption of non-diet soda with BFFQ responses to fat intake variables, cholesterol, and daily servings of whole grains, breads, cereals, rice, and pastas show that this particular question is related to multiple dietary behaviors and not just the consumption of beverages with added sugar. The HEVS questions, however, can help launch a dialogue between patients and providers to explore these interactions, which are often unique and individualized.

Though the Cronbach's alpha did not reach the common cutoff of 0.7, the alpha we obtained, which was >0.40, indicated acceptable consistency through the tested item [17]. Future factor analysis, which would require a larger sample size, would help to combine items into a few factors in order to obtain higher consistency.

Previous evaluation supported the content validity of this instrument, associating responses of the HEVS to weight [14]. Our sample for this study was too small to determine such associations.

The HEVS assesses the frequency of eating patterns by asking respondents the number of times per day and week particular foods are ingested. This differs from most food frequency questionnaires, which ask respondents to quantify servings of food. Often, the concept of serving size is difficult for people (patients and providers alike) to quantify. Communicating the frequency of a dietary behavior is less complex. Traynor et al. found no significant difference between responses of fruit and vegetable consumption quantified by servings or frequency (*P* = 0.94) [18]. The investigators created the HEVS in agreement with the findings of this investigation.

This study had a small sample size, limiting its power. However, we would anticipate stronger correlation between the HEVS and FFQ given a larger study population. Furthermore, causation could not be established with this survey study. Other limitations of this study include the following. We collected data for this study during spring and summer months; therefore, we could not account for seasonal variations. The data were skewed according to gender (female = 56 and male = 4); therefore, we could not categorize our analysis by gender. The gender and ethnic/racial diversity of our sample was limited and did not match that of the general US population. Furthermore, the administration method made this study subject to recall bias, and the setting might introduce other subject biases, as we used a self-administered questionnaire collected in a clinic environment.

This study utilized the responses from staff working in primary care medical clinics. They all had at least a high school education (or educational equivalent) and 1-2 years of vocational training. We did not collect specific data on level of education, and we have inadequate male representation. We assumed responses from our population would closely match those of the general adult population of Utah. However, this assumption is not scientifically based and limits the generalizability of this study. Future endeavors could assess a patient population to allow further inferences.

Our ultimate goal is to create an effective screening tool to identify key eating behaviors that may assist primary care providers with managing excessive weight. We envision a concise 3–5 item tool, feasible for use during a 15-minute clinic visit. With the emergence of electronic medical records, the questionnaire can be programmed in with nursing notes or the vital signs. The nurse or medical assistant can ask and record the responses while rooming a patient, or the provider can do the same as they encounter the patient. Alternatively, the questionnaire can be administered in paper form, and patients can respond as they wait for the provider in the reception area or exam room. 

We consider this to be a successful investigation for the criterion validity of the HEVS. Medical management of weight in clinical settings is centered upon (1) assessment and (2) counseling based upon that assessment. With the HEVS, providers can assess eating behaviors and identify patients who may need counseling to prevent or manage overweight and/or obesity. Our next endeavor will be to create a counseling instrument that complements the HEVS, then pilot the use of these instruments in clinical settings.

## Figures and Tables

**Table 1:** Summary of participant demographics.

Characteristic	Frequency (*N* = 60)	Percent
Gender		
Female	56	93.3
Male	4	6.7
BMI category		
Normal (BMI 19–24)	19	31.7
Overweight (BMI 25–29)	15	25.0
Obese (BMI > 30)	26	43.3
Race		
Latino/a or Hispanic	15	25.4
Caucasian	32	54.2
Native American	1	1.7
No response	11	18.6
Job		
Billing	1	1.7
Building Maintenance	1	1.7
Clinical Care Coordinator	7	11.7
Clinic Director Assistant	1	1.7
Scheduler	5	8.3
Lab Technician	1	1.7
Licensed Practicing Nurse	1	1.7
Manager	1	1.7
Medical Assistant	33	55.0
Medical Records	2	3.3
Nurse Case Manager	1	1.7
Pharmacy Coordinator	1	1.7
Program Assistant	1	1.7
Registered Nurse	4	6.7

**Table 2:** Descriptive summary of responses to HEVS and BFFQ, *N* = 60.

Questionnaire items	Mean	Std Dev^∗^	Minimum	Maximum
HEVS				
Restaurant/fast food consumption last week	2.3	1.9	0	7
Typical restaurant/fast food consumption	2.2	1.8	0	7
Nondiet soda consumption yesterday	0.4	0.8	0	4
Typical nondiet soda consumption	0.4	0.6	0	2
Juice/punch consumption yesterday	0.5	1.0	0	5
Typical juice/punch consumption	0.8	1.2	0	7
Vegetable consumption yesterday	1.6	1.3	0	5
Typical vegetable consumption	1.7	1.0	0	5
Fruit consumption yesterday	1.6	1.4	0	6
Typical fruit consumption	1.9	1.2	0	6
BFFQ
Food energy (kcals^†^)	1671.0	752.1	504.3	4602.0
Fat (gms^‡^)	65.7	31.7	19.9	169.0
Saturated fat (gms)	20.5	9.7	5.6	48.6
Monounsaturated fatty acids (gms)	26.1	13.8	7.3	75.8
Cholesterol, mg	196.8	105.2	51.3	560.5
Dietary fiber (gms)	15.7	7.6	5.1	46.3
Sugars, total (gms)	96.3	55.3	17.4	336.7
Trans fats, total (gms)	2.4	1.3	0.7	5.5
% of kcal from sweets, desserts	15.0	9.3	3.0	38.8
% carbohydrate kcals	50.0	6.8	38.6	64.9
Daily svgs^§^ breads, cereals, rice, pasta	5.0	3.0	1.0	14.5
Daily svgs fats and oils, sweets, sodas	3.3	1.7	0.7	8.6
Average daily svgs of whole grains	0.9	0.9	0.0	4.4
Dietary fiber from beans (gms)	2.8	2.6	0.2	11.8
Dietary fiber from vegetables, fruits (gms)	5.7	3.9	1.3	26.1
Daily svgs of vegetables	2.2	1.8	0.4	12.0
Daily frequency of fruits and fruit juices	1.1	0.8	0.3	3.2

^∗^Std Dev: standard deviation.

^†^kcal: kilocalorie.

^‡^gms: grams.

^§^svgs: servings.

**Table 3:** Discriminant and criterion Pearson correlation coefficients between Healthy Eating Vital Sign and Block Food Frequency Questionnaire, *N* = 60.

	Healthy Eating Vital Sign Questions^†^									
	1	2	3	4	5	6	7	8	9	10
BFFQ										
Energy (kcals)	0.2	0.2	0.2	0.4^∗∗^	0.3^∗^	0.3^∗^	−0.0	−0.1	0.0	−0.1
Fat (gms)	0.3^∗^	0.3	0.3^∗^	0.4^∗∗^	0.2	0.2	−0.1	−0.1	−0.1	−0.1
Saturated fat (gms)	0.3^∗^	0.2	0.3^∗^	0.4^∗∗^	0.3^∗^	0.3^∗^	−0.10	−0.2	−0.1	−0.1
Monounsaturated fat (gms)	0.4^∗∗^	0.3^∗^	0.3^∗^	0.4^∗∗^	0.2	0.2	−0.1	−0.1	−0.1	−0.2
Cholesterol (mg)	0.3	0.3	0.2	0.4^∗∗^	0.3^∗^	0.3^∗^	−0.2	−0.2	−0.1	−0.1
Fiber (gms)	0.0	−0.0	0.0	0.1	0.1	0.0	0.3^∗^	0.3^∗^	0.3^∗^	0.1
Sugar (gms)	0.0	0.0	0.0	0.4^∗∗^	0.4^∗∗∗^	0.3^∗^	−0.0	−0.1	0.1	0.1
Trans-fats (gms)	0.5^∗∗∗^	0.4^∗^	0.2	0.4^∗∗^	0.2	0.2	−0.2	−0.3^∗^	−0.2	−0.2
Sweets/desserts (%kcals)	0.1	0.3	0.1	0.3^∗^	0.1	0.1	−0.1	−0.2	−0.1	−0.1
Breads, cereals, rice, and pasta (svgs)	0.4^∗∗^	0.3^∗^	0.2	0.3^∗^	0.2	0.2	−0.1	−0.1	−0.1	−0.2
Fats and oils, sweets, and sodas (svgs)	0.2	0.2	0.3^∗^	0.4^∗∗^	0.2	0.1	−0.1	−0.2	−0.2	−0.1
Whole grains (svgs)	0.0	−0.0	0.1	0.3^∗^	−0.0	0.0	0.1	−0.0	0.0	−0.1
Fiber beans (gms)	−0.0	−0.0	0.0	0.2	0.2	0.1	0.4	0.4^∗∗^	0.3^∗^	0.3
Fiber vegetables, fruits (gms)	−0.1	−0.2	−0.2	−0.2	−0.1	−0.1	0.4^∗∗^	0.4^∗∗^	0.3^∗^	0.3
Vegetables (svgs)	−0.1	−0.1	−0.1	−0.2	−0.1	−0.1	0.3^∗^	0.3^∗^	0.3	0.2
Fruits and fruit juices (frqncy)	−0.1	−0.2	−0.1	−0.1	0.2	0.1	0.4^∗∗^	0.3^∗^	0.4^∗∗^	0.4^∗∗^

*P < .05.

**P < .01.

***P < .001.

^†^HEVS 1: restaurant/fast food consumption last week. HEVS 2: typical restaurant/fast food consumption. HEVS 3: non diet soda consumption yesterday. HEVS 4: typical nondiet soda consumption. HEVS 5: juice/punch consumption yesterday. HEVS 6: juice/punch consumption yesterday. HEVS 7: vegetable consumption yesterday. HEVS 8: typical vegetable consumption. HEVS 9: fruit consumption yesterday. HEVS 10: typical fruit consumption.

**Table 4:** Internal consistency of HEVS questions.

Variables	Cronbach coefficient alpha
Raw	0.45
Standardized	0.49

**Table 5:** The Healthy Eating Vital Signs (HEVS).

Question	Answer
How many times did you eat restaurant or fast food last week? (For example Chili's, McDonald's, Burger King, etc.)	0 1 2 3 4 5 6 7 more than 7
How many times do you typically eat restaurant or fast food in *one week* (7 days)?	0 1 2 3 4 5 6 7 more than 7
How many cans of non-diet soda pop did you drink yesterday? (For example Coke, Pepsi, Sprite)	0 1 2 3 4 5 6 7 more than 7
How many cans do you typically drink non-diet soda pop in *one day*?	0 1 2 3 4 5 6 7 more than 7
How many times did you drink juice or punch yesterday? (For example orange juice, apple juice, Sunny Delight)	0 1 2 3 4 5 6 7 more than 7
How many times do you typically drink juice or punch in *one day*?	0 1 2 3 4 5 6 7 more than 7
How many times did you eat vegetables yesterday? (For example broccoli, spinach, greens, salad, etc.)	0 1 2 3 4 5 6 7 more than 7
How many times do you typically eat vegetables in *one day*?	0 1 2 3 4 5 6 7 more than 7
How many times did you eat fruit yesterday? (For example an apple, an orange, a hand full of grapes, etc.)	0 1 2 3 4 5 6 7 more than 7
How many times do you typically eat fruit in *one day*?	0 1 2 3 4 5 6 7 more than 7
How many times do you typically eat breakfast in *one week* (7 days)?	0 1 2 3 4 5 6 7
When eating restaurant food or fast food, do you eat all of the food served to you at one time?	Never ∣ Rarely ∣ Occasionally ∣ Sometimes ∣ Often ∣ Usually ∣ Always

## Appendix

For more details See Table 5.

## References

[1] U.D.o.H.a.H. Services Objective 19-2. Reduce the proportion of adults who are obese.

[2] (2010). Vital signs: state-specific obesity prevalence among adults—United States, 2009. *Morbidity and Mortality Weekly Report*.

[3] Flegal KM, Carroll MD, Ogden CL, Curtin LR (2010). Prevalence and trends in obesity among US adults, 1999–2008. *Journal of the American Medical Association*.

[4] Nawaz H, Katz DL (2001). American college of preventive medicine practice policy statement: weight management counseling of overweight adults. *American Journal of Preventive Medicine*.

[5] Potter MB, Vu JD, Croughan-Minihane M (2001). Weight management: what patients want from their primary care physicians. *Journal of Family Practice*.

[6] Galuska DA, Will JC, Serdula MK, Ford ES (1999). Are health care professionals advising obese patients to lose weight?. *Journal of the American Medical Association*.

[7] Must A, Spadano J, Coakley EH, Field AE, Colditz G, Dietz WH (1999). The disease burden associated with overweight and obesity. *Journal of the American Medical Association*.

[8] Block G, Hartman AM, Dresser CM, Carroll MD, Gannon J, Gardner L (1986). A data-based approach to diet questionnaire design and testing. *American Journal of Epidemiology*.

[9] Block G, Coyle LM, Hartman AM, Scoppa SM (1994). Revision of dietary analysis software for the health habits and history questionnaire. *American Journal of Epidemiology*.

[10] Glasgow RE, Ory MG, Klesges LM, Cifuentes M, Fernald DH, Green LA (2005). Practical and relevant self-report measures of patient health behaviors for primary care research. *Annals of Family Medicine*.

[11] Rosamond WD, Ammerman AS, Holliday JL (2000). Cardiovascular disease risk factor intervention in low-income women: the North Carolina WISEWOMAN project. *Preventive Medicine*.

[12] Toobert DJ, Hampson SE, Glasgow RE (2000). The summary of diabetes self-care activities measure: results from 7 studies and a revised scale. *Diabetes Care*.

[13] Greenwood JLJ, Stanford JB (2008). Preventing or improving obesity by addressing specific eating patterns. *Journal of the American Board of Family Medicine*.

[14] Greenwood JLJ, Murtaugh MA, Omura EM, Alder SC, Stanford JB (2008). Creating a clinical screening questionnaire for eating behaviors associated with overweight and obesity. *Journal of the American Board of Family Medicine*.

[15] Nielsen SJ, Popkin BM (2003). Patterns and trends in food portion sizes, 1977–1998. *Journal of the American Medical Association*.

[16] Block G, Woods M, Potosky A, Clifford C (1990). Validation of a self-administered diet history questionnaire using multiple diet records. *Journal of Clinical Epidemiology*.

[17] Sprotles GB, Kendall EL (1986). A methodology for profiling consumers' decision-making style. *Journal of Consumer Affairs*.

[18] Traynor MM, Holowaty PH, Reid DJ, Gray-Donald K (2006). Vegetable and fruit food frequency questionnaire serves as a proxy for quantified intake. *Canadian Journal of Public Health*.

